# End-of-Life Care for the Incarcerated: A Case Study on Privatization

**DOI:** 10.1093/geroni/igaf122.201

**Published:** 2025-12-31

**Authors:** Hannah Schwendeman

**Affiliations:** University of Minnesota, Minneapolis, Minnesota, United States

## Abstract

The U.S. prison population is rapidly aging as an unprecedented number of older adults live and die behind bars. Due to tough-on-crime policies that lengthened sentences, the population of older adults in prison has increased nearly 400% in the last 20 years. In response to this aging crisis, state administrators are building new prison hospice units, contracting with private healthcare companies, and training both staff and incarcerated people to care for older prisoners. In this paper, I ask: what logics and practices are used by prison administrators and public officials to provide end-of-life care for incarcerated people? Through legal archival analysis, I analyze how state actors are responding to the increasing need for end-of-life care for incarcerated people. Specifically, I analyze how the state of Connecticut, the first state to receive Medicaid funding for this population, privatized end-of-life care for the incarcerated and the legal challenges that ensued.

